# Glibenclamide—10-h Treatment Window in a Clinically Relevant Model of Stroke

**DOI:** 10.1007/s12975-012-0149-x

**Published:** 2012-03-07

**Authors:** J. Marc Simard, Seung Kyoon Woo, Natalia Tsymbalyuk, Oksana Voloshyn, Vladimir Yurovsky, Svetlana Ivanova, Ryan Lee, Volodymyr Gerzanich

**Affiliations:** 1 Department of Neurosurgery, University of Maryland School of Medicine, 22 S. Greene Street, Suite S12D, Baltimore, MD 21201-1595 USA; 2 Department of Pathology, University of Maryland School of Medicine, Baltimore, MD 21201 USA; 3 Department of Physiology, University of Maryland School of Medicine, Baltimore, MD 21201 USA

**Keywords:** Cerebral ischemia, Stroke, Glibenclamide, Sur1, Sur1-regulated NC_Ca-ATP_ channel, Recombinant tissue plasminogen activator, Rat

## Abstract

Glibenclamide improves outcomes in rat models of stroke, with treatment as late as 6 h after onset of ischemia shown to be beneficial. Because the molecular target of glibenclamide, the sulfonylurea receptor 1 (Sur1)-regulated NC_Ca-ATP_ channel, is upregulated de novo by a complex transcriptional mechanism, and the principal pathophysiological target, brain swelling, requires hours to develop, we hypothesized that the treatment window would exceed 6 h. We studied a clinically relevant rat model of stroke in which middle cerebral artery occlusion (75% < reduction in LDF signal ≤90%) was produced using an intra-arterial occluder. Recanalization was obtained 4.5 h later by removing the occluder. At that time, we administered recombinant tissue plasminogen activator (rtPA; 0.9 mg/kg IV over 30 min). Immunolabeling showed modest expression of Sur1 5 h after onset of ischemia, with expression increasing 7- to 11-fold (*P* < 0.01) by 24 h. Rats were administered either vehicle or glibenclamide (10 μg/kg IP loading dose plus 200 ng/h by constant subcutaneous infusion) beginning 4.5 or 10 h after onset of ischemia. In rats treated at 4.5 or 10 h, glibenclamide significantly reduced hemispheric swelling at 24 h from (mean ± SEM) 14.7 ± 1.5% to 8.1 ± 1.6% or 8.8 ± 1.1% (both *P* < 0.01), respectively, and significantly reduced 48-h mortality from 53% to 17% or 12% (both *P* < 0.01), and improved Garcia scores at 48 h from 3.8 ± 0.62 to 7.6 ± 0.70 or 8.4 ± 0.74 (both *P* < 0.01). We conclude that, in a clinically relevant model of stroke, the treatment window for glibenclamide extends to 10 h after onset of ischemia.

## Introduction

Glibenclamide confers protection in various rat models of stroke, including non-lethal models of thromboembolic, permanent and temporary (105 min) occlusion, as well as models of malignant cerebral edema [21, 23, 25]. In these models, protection is manifested as significant improvements in edema, brain swelling, lesion size, white matter preservation, neurological function and mortality. Beneficial effects of glibenclamide have been reported independently using in vitro and in vivo models of ischemia/hypoxia [15, 18].

In cerebral ischemia, the molecular target of glibenclamide is the sulfonylurea receptor 1 (Sur1)-regulated NC_Ca-ATP_ channel. All members of the neurovascular unit upregulate the Sur1-regulated NC_Ca-ATP_ channel after ischemia. However, its role in endothelial cell swelling, which contributes to ischemia, and its role in dysfunction of inter-endothelial tight junctions, which contributes to edema formation and brain swelling, are particularly important for many of the beneficial effects observed from blocking the channel.

The Sur1-regulated NC_Ca-ATP_ channel is not constitutively expressed but is transcriptionally upregulated after onset of ischemia. Recent work showed that the molecular mechanism responsible for transcriptional upregulation of the channel in endothelium involves a two-step sequential gene activation process [30]. Hypoxia first activates hypoxia inducible factor 1, which results in transcriptional upregulation of the transcription factor, specificity protein 1 (Sp1). Upregulation of Sp1, in turn, results in transcriptional upregulation of Sur1, which then goes on to form functional ion channels that are transported to and inserted into the cell membrane. This multistep mechanism involving sequential gene activation, channel assembly and transport predicts that a prolonged time would be required before the molecular target of glibenclamide is in place. Moreover, the principal pathophysiological target of glibenclamide, brain swelling, requires hours to develop [13, 20]. As a result, we hypothesized that the treatment window for glibenclamide would exceed 6 h.

Here, we tested the hypothesis that glibenclamide would have a long treatment window in a clinically relevant rat model of stroke. Middle cerebral artery occlusion (MCAo) was produced using an intra-arterial occluder. Recanalization was obtained by removing the occluder at 4.5 h, simulating intra-arterial mechanical clot retrieval [6]. At the same time, we administered rtPA at the latest time and at the dose used in humans (0.9 mg/kg IV) [2, 9]. Briefly, we found that this model was associated with high mortality and severe neurological disability, and that glibenclamide administered at either 4.5 or 10 h after onset of ischemia significantly improved outcomes.

## Methods

### Rat Model of Stroke

All surgical procedures were approved by the Institutional Animal Care and Use Committee of the University of Maryland. Male Wistar rats (250–275 g; Harlan, Indianapolis, IN) were anesthetized (ketamine, 60 mg/kg, and xylazine, 7.5 mg/kg, IP) and allowed to breath spontaneously room air supplemented with oxygen to maintain 90% < sO_2_ < 99% by pulse oximetry. The procedures for intra-arterial MCAo via the cervical external carotid artery (ECA), for monitoring laser Doppler flowmetry (LDF) signals over the involved cortex, for monitoring blood gases, for implanting mini-osmotic pumps with catheters for delayed start of infusion, for post-operative recovery of the animals and for necropsy to establish stroke-related death, have been described [23, 25]. Compared to our previous reports, the principal differences in the present study were that: (1) we used commercially available intra-arterial occluders (0.39 mm; catalogue #4039PK5Re; Doccol Corp, Redlands CA); (2) the rats were re-anesthetized at ~4 h to acquire IV access via the external jugular vein, to remove the occluder at 4.5 h, to ligate the stump of the ECA, and to implant a mini-osmotic pump subcutaneously; (3) after removing the occluder, an infusion of rtPA [Cathflo Activase (Alteplase), Genetech, Inc., San Francisco, CA], 0.9 mg/kg IV over 30 min, was started; (4) at the time designated for treatment (4.5 or 10 h after onset of ischemia), rats received either vehicle or glibenclamide (10 μg/kg IP loading dose plus start of constant subcutaneous infusion of 200 ng/h for 48 h). The low dose of glibenclamide used here has been shown repeatedly not to cause hypoglycemia [21, 24, 25].

Blood gases immediately prior to MCAo were (mean ± SD): pO_2_, 147 ± 18 mmHg; pCO_2_, 56 ± 1 mmHg and pH, 7.29 ± 0.01; glucose, 135 ± 7; Hct, 46 ± 1; sO_2_, 97 ± 1; pO_2_, 149 ± 23 mmHg; pCO_2_, 57 ± 1 mmHg and pH, 7.30 ± 0.01; glucose, 126 ± 6; Hct, 46 ± 1; sO_2_, 96 ± 1; for vehicle vs. glibenclamide treated rats, respectively.

LDF signals were monitored during the 30 min following positioning of the MCA occluder. For the three groups (vehicle, 4.5- and 10-h glibenclamide), LDF signals were reduced by (mean ± SEM): 84.5 ± 0.7%, 81.6 ± 2.1%, and 83.7 ± 1.2%, respectively, which were not significantly different from each other (by analysis of variance [ANOVA], *P* = 0.3).

### Drug Formulation and Pump Preparation

A stock solution of glibenclamide was prepared by placing 25 mg glibenclamide (#G2539; meets USP testing; Sigma, St. Louis, MO) into 10 ml dimethyl sulfoxide (DMSO). The solution to be loaded into the mini-osmotic pumps (Alzet 2001, 1.0 μl/h; Alzet Corp., Cupertino, CA) was made by taking 2.3 ml unbuffered normal saline (NS), adding 4 μl of 10 N NaOH (undiluted Fixanal; Riedel-deHaën, Seelze, Germany), then adding 200 μl stock solution, in that order to prevent precipitation of drug. The solution to be used for the loading dose was made by adding 4 μl of stock solution to 1 ml NS. For vehicle controls, solutions were made with DMSO, NS and NaOH, as above, but glibenclamide was omitted. The pumps were filled with a measured volume of solution (222–238 μl), with the volume determined by the difference in mass before and after loading. After loading, the pumps were primed overnight in NS at 37°C with the outlet of the pump connected to a length of PE60 tubing that extended above the level of the priming solution, to prevent H^+^ ions from entering the pump chamber. Prior to implanting, the outlet of the pump either was left unconnected, for immediate start of drug infusion, or it was connected to an empty catheter (PE60 tubing), 5.5 μl in volume, to obtain a 5.5-h delay in the start of drug infusion [25].

During the course of these experiments, periodic checks were carried out to confirm proper loading and functioning of the pumps. At the end of an experiment (after stroke-related death or 48 h), the pump was removed from the animal and function was assessed by assuring proper seating of the cap, and by measuring residual pump volume. Proper formulation and correct loading were assessed by measuring the glibenclamide concentration in the residual solution. The concentration of glibenclamide in the pump (nominally 200 μg/ml) was measured spectrophotometrically (absorbance at 239 nm) [8] and was compared to a standard solution prepared at the same time and stored in a glass vial. In one instance, these checks led to the exclusion of one rat when it was found that the pump had not been properly loaded.

### Stability of Glibenclamide Formulation In Vivo

Experiments were carried out to assess the solubility of glibenclamide (200 μg/ml in 8% DMSO/NS) at pH 6–8. Solutions were prepared, centrifuged (15,000 × *g* for 10 min) and the glibenclamide concentration of the solution was determined spectrophotometrically (absorbance at 239 nm).

Experiments were carried out with five uninjured rats to determine the stability of glibenclamide solutions in vivo. Glibenclamide solutions (2.5 ml of 200 μg/ml in 8% DMSO/NS) were prepared using 1, 2 or 4 μl of 10 N NaOH (undiluted Fixanal). These solutions were loaded into pumps. After priming (with the outlet protected as above), the pumps (one or two per rat) were implanted. At periodic intervals, pumps were removed from the animals and the concentration of glibenclamide in the pump solution was measured.

### Sample Size Calculation for the Model of Stroke

Initial experiments with the model used here suggested that mortality rates of ~50% and ~20% likely would be encountered with vehicle vs. glibenclamide treatment. Power analysis was performed (Power and Precision; release 3.2) for a two-sample proportion with these rates. It was found that group sizes of 25–40 would yield 61–82% power of rejecting the null hypothesis (*α* = 0.05; two-tailed).

### Experimental Series and Study Groups

In Series 1, 13 rats underwent MCAo plus rtPA administration as above; five rats were euthanized at 5 h after onset of ischemia (the end of the 30-min infusion of rtPA) and eight rats were euthanized 24 h after onset of ischemia; these brains were used for immunolabeling experiments. In Series 2, 46 rats underwent MCAo plus rtPA administration as above; 18 rats were administered vehicle and 18 rats were administered glibenclamide at 4.5 h after onset of ischemia (the time of recanalization); ten rats were administered glibenclamide at 10 h after onset of ischemia (5.5 h after recanalization). Of the 36 rats treated at 4.5 h, 16 (eight vehicle and eight glibenclamide) were euthanized at 10 h, and the remainder were euthanized at 24 h. Of the rats treated at 10 h, all were euthanized at 24 h. All of these brains were used to assess hemispheric swelling. In Series 3–5, rats underwent MCAo plus rtPA administration as above; these rats were used to examine the effect of vehicle vs. glibenclamide administered at different times (4.5 or 10 h after onset of ischemia) on preclinical outcomes (mortality, Garcia scores, infarct size at 48 h). In Series 3 (vehicle vs. 4.5-h glibenclamide), Series 4 (vehicle vs. 10-h glibenclamide), and Series 5 (vehicle vs. 4.5-h glibenclamide), there were 55, 50, and 40 rats, respectively. In each of these series, the allocation ratio was ~1 vehicle to 2 glibenclamide. All series were carried out by two surgeons performing the same experiment in tandem, which helped to control for surgeon variability. The 4.5-h experiment of Series 3 was repeated after several months as Series 5, which helped to control for temporal variability. No effect of administering vehicle at 4.5 vs. 10 h was uncovered, allowing data from all the vehicle-treated rats to be pooled.

### Exclusions

Rats were not enrolled if LDF signals did not show sustained reduction of 75–90% for the 30-min period of monitoring, or if the sustained reduction exceeded 90%. The latter criterion was implemented due to the exceedingly high incidence of early death associated with >90% reduction. Of the 145 rats in Series 3–5, 130 met the LDF inclusion criteria. Of the 130 eligible rats, 13 were excluded after treatment allocation as follows (numbers in parentheses denote the number of rats from the vehicle, 4.5-h, and 10-h glibenclamide groups, respectively): subarachnoid hemorrhage (1,2,0), subdural hemorrhage (1,0,0), premature death before treatment (2,2,0), no discernable infarct (2,1,0), bilateral infarct (0,1,0), technical problem with the treatment (0,0,1). Final group sizes were: 51, 41, 25 for vehicle, 4.5-h, and 10-h glibenclamide treatment, respectively.

### Good Laboratory Practice

Care was taken to follow good laboratory practice. Daily treatment allocation (vehicle vs. glibenclamide) was determined by one investigator (VG) using coin toss, except when the need arose to balance enrollment. Another investigator (SKW) prepared and coded the pumps and syringes and maintained the log of treatments for use later in decoding, but did not otherwise participate. Two surgeons (NT and OV), both blinded to treatment, independently performed surgeries, but did not participate in outcome evaluations. Preclinical outcomes (mortality, Garcia scores and necropsies) were determined by a separate investigator (VY) who was blinded to treatment. Measurements of TTC-lesion sizes and hemispheric swelling were performed by another investigator (SI) who was blinded to treatment.

### Immunohistochemistry

Rats from Series 1 were used for immunohistochemistry. After transcardiac perfusion/fixation with 10% neutral buffered formalin and cryoprotection of the brain with 30% sucrose, cryosections were immunolabeled using goat anti-Sur1 antibody (1:200; SC-5789; Santa Cruz Biotechnology, Santa Cruz, CA) and, in some cases, co-labeled with laminin (1:200; FITC-conjugated; EY Laboratories, San Mateo CA) to identify microvessels. Alexa Fluor-555-conjugated, species appropriate secondary antibody (Invitrogen, Carlsbad, CA) was used for visualization. Omission of primary antibody was used as a negative control. The sections were coverslipped with polar mounting medium containing anti-fade reagent and the nuclear dye, 4′,6′ diamino-2-phenylindole (DAPI) (Invitrogen) and were examined using epifluorescence microscopy. Previous studies from this laboratory confirmed the absence of labeling in control tissues, and the specificity of the antibody against Sur1 used here [21, 23, 25].

### Quantitative Immunohistochemistry

**Fig. 1:**
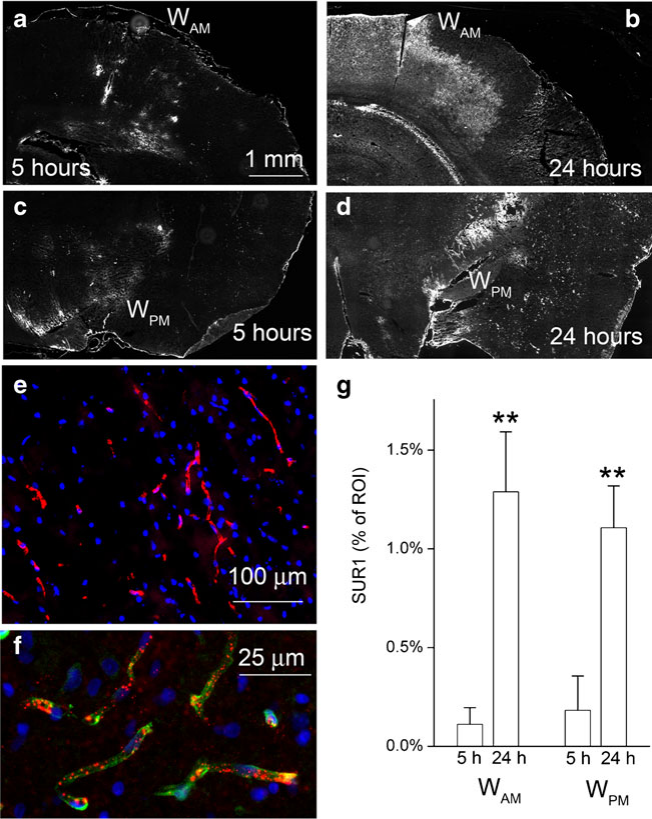
Sulfonylurea receptor 1 (*Sur1*) continues to be upregulated after recanalization. **a**–**d** Coronal sections of rat brains immunolabeled for Sur1 5 h (**a**, **c**) or 24 h (**b**, **d**) after onset of ischemia; note the immunolabeling for Sur1 in the watershed region of the anterior and middle cerebral arteries (*W*_*AM*_) (**a**, **b**) and the watershed region of the posterior and middle cerebral arteries (*W*_*PM*_) (**c**, **d**) that increased with time. **e**, **f** Immunolabeling of W_PM_ region 24 h after onset of ischemia shows prominent upregulation of Sur1 (*red*) in elongated structures that co-label for laminin (*green*), consistent with microvessels; nuclei labeled with DAPI (*blue*). **g** Quantitative immunohistochemistry showing large increases in Sur1 expression in W_AM_ and W_PM_ regions that developed between 5 and 24 h after onset of ischemia; five and eight rats, respectively; **P < 0.01. For all experiments, MCAo was obtained using an intra-arterial occluder that was left in place for 4.5 h, after which a 30-min infusion of rtPA (0.9 mg/kg IV) was started

Unbiased measurements of signal intensity within regions of interest (ROI) were obtained using NIS-Elements AR software (Nikon Instruments, Melville, NY) from sections immunolabeled in a single batch, as previously described [7, 22]. All ROI images for a given signal were captured using uniform parameters of magnification, area, exposure, and gain. Segmentation analysis was performed by computing a histogram of pixel intensity for a particular ROI. For SUR1, specific labeling was defined as pixels with signal intensity greater than 2× that of background, and the area occupied by pixels with specific labeling was used to determine the percent area with specific labeling (% ROI). ROIs consisted of rectangular areas, 2.5×6.0 mm that were positioned to encompass the watershed region of the anterior and middle cerebral arteries (W_AM_) and the watershed region of the posterior and middle cerebral arteries (W_PM_) (as delineated in Fig. 1a–d).

### Hemispheric Swelling at 10 and 24 h

Rats from Series 2 were used to assess swelling. For rats that died, the skull containing the brain was immersed in formalin for 3 days. Survivors were euthanized at 10 or 24 h, perfused with NS then formalin, and the skull containing the brain was immersed in formalin for 3 days. Subsequently, 2-mm coronal sections of the fixed brains were prepared and imaged using a flatbed scanner. Images of six consecutive slices were analyzed using Photoshop (Adobe) to obtain area measurements of both hemispheres, which were used to compute hemispheric swelling in percent, a robust measure independent of the fixation procedure [17].

### Functional Outcome

Neurological function was assessed using the modified Garcia score [19], with the only modification being that a score of 0 was assigned for death. Scores were determined at 24 and 48 h after onset of ischemia. Animals that survived were euthanized at 48 h.

### Infarct Volume

For both rats that succumbed to stroke-related death as well as those euthanized at 48 h, brains were evaluated to obtain corrected infarct volumes. Coronal sections, 2 mm thick, were immersed in 2% 2,3,5-triphenyltetrazolium chloride (TTC) (Sigma-Aldrich, USA) in NS for 20 min at 37°C. The TTC-stained sections were imaged using a flatbed scanner. Images of five consecutive slices were analyzed using Photoshop (Adobe) to obtain area measurements of the TTC-negative region, and of the ipsilateral and contralateral TTC-positive regions, which were used to compute the “corrected” hemispheric infarct volume in percent, a robust measure independent of the processing procedure [17].

### Statistical Analysis

Calculations were performed using either InStat, version 3.1 (GraphPad Software, Inc.) or OriginPro8 (OriginLab Corp.). Expression levels of Sur1 at 5 vs. 24 h were analyzed using Student’s *t*-test. Rates of mortality and of petechial hemorrhages were analyzed using a 2 × 2 contingency table with Fisher’s exact test. Hemispheric swelling for the four groups of rats treated at 4.5 h was analyzed using a two-way ANOVA with the Bonferroni test. Hemispheric swelling for the single group treated with glibenclamide at 10 h was compared to swelling in groups treated at 4.5 h using Student’s *t*-test. Garcia scores were compared between groups using the Kruskal–Wallis test with Dunn’s post-hoc comparisons. The change in Garcia scores of individual rats between 24 and 48 h was analyzed using a Wilcoxon matched-pairs test. TTC-lesion volumes were analyzed using a one-way ANOVA with Bonferroni post-hoc comparisons. The correlations between LDF signal and Garcia score, and between infarct volume and Garcia score were analyzed using the Spearman rank correlation.

## Results

### Sur1 Upregulation

Sur1 was previously shown to be upregulated in various rat models of stroke [21, 23, 25], but not in the model studied here with a prolonged time before reperfusion. The study of tissues harvested 5 h after onset of ischemia (after completing the IV infusion of rtPA) showed modest immunolabeling for Sur1 in the watershed region of the anterior and middle cerebral arteries (W_AM_) (Fig. 1a), as well as in the watershed region of the posterior and middle cerebral arteries (W_PM_) (Fig. 1c). Upregulation of Sur1 in watershed regions is typical [21]. Immunolabeling tissues harvested 24 h after onset of ischemia showed extensive necrosis in the MCA cortex (upregulation of Sur1 is not observed in necrotic tissues [21]), as well as prominent upregulation of Sur1 in both watershed regions (Fig. 1b, d). As previously reported [21], much of the upregulation in watershed regions localized to microvessels (Fig. 1e, f). Quantification showed that, in both watershed regions, Sur1 expression at 24 h was 7- to 11-fold greater than at 5 h (Fig. 1g), consistent with continued upregulation of Sur1 after recanalization.

### Formulation of Glibenclamide

**Fig. 2:**
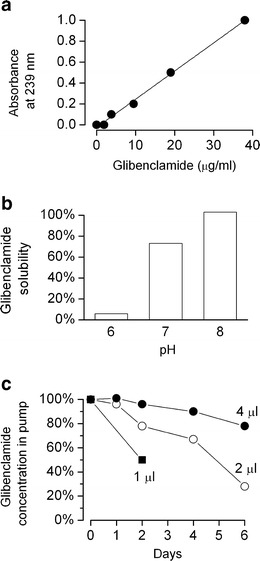
Glibenclamide formulation. **a** Spectrophotometric measurement shows that absorbance at 239 nm is linearly related to the concentration of glibenclamide solubilized in normal saline supplemented with 8% dimethylsulfoxide (*DMSO*); absorbance due to DMSO was subtracted. **b** The solubility of glibenclamide, prepared as a 200 μg/ml solution in NS supplemented with 8% DMSO, depends on pH. **c** The stability over time of glibenclamide solutions (2.5 ml of 200 μg/ml in 8% DMSO/NS plus 1, 2, or 4 μl of 10 N NaOH) placed inside of an implanted mini-osmotic pump depends on the amount of buffering capacity included in the formulation; buffering capacity supplied by the volume of 10 N NaOH indicated; in **b** and **c**, spectrophotometric measurements were made after a 10-fold dilution

Glibenclamide (adopted US name, glyburide, p*K*_a_ = 5.3) is a weak acid that is sparingly soluble in water, but whose solubility increase with pH. We measured the solubility of glibenclamide spectrophotometrically (absorbance at 239 nm; Fig. 2a) [8] at pH 6–8, when solutions were prepared at a concentration of 200 μg/ml in NS containing 8% of the co-solvent, DMSO. Even with the co-solvent, pH 8 was required to achieve 100% solubility (Fig. 2b).

A drug solution at high pH loaded into a pump implanted into an animal is predicted to drift toward neutral pH as H^+^ ions from the body enter the drug solution, resulting in drug precipitating out of solution. The loss of high pH can be rapid, since H^+^ ions are transported though water at a rate ~5 times faster than that of other cations whose diffusion is governed solely by thermal motion [3, 12]. Preventing the loss in high pH requires sufficient buffering capacity in the pump solution, in this case an excess of OH^−^ ions, to maintain a stable drug concentration.

We examined the stability of solutions of glibenclamide inside of pumps implanted into uninjured rats. We found that a solution of glibenclamide (2.5 ml of 200 μg/ml in 8% DMSO/NS) prepared with 1 or 2 μl of 10 N NaOH (pH 11.5 or 12, respectively) exhibited a progressive decline in concentration (Fig. 2c, filled squares, empty circles). By contrast, a solution prepared with 4 μl of 10 N NaOH (pH 12.3) was stable for 2 days and lost ~20% over 6 days (Fig. 2c, filled circles). The latter formulation was used for the preclinical experiments described below.

### Swelling

**Fig. 3:**
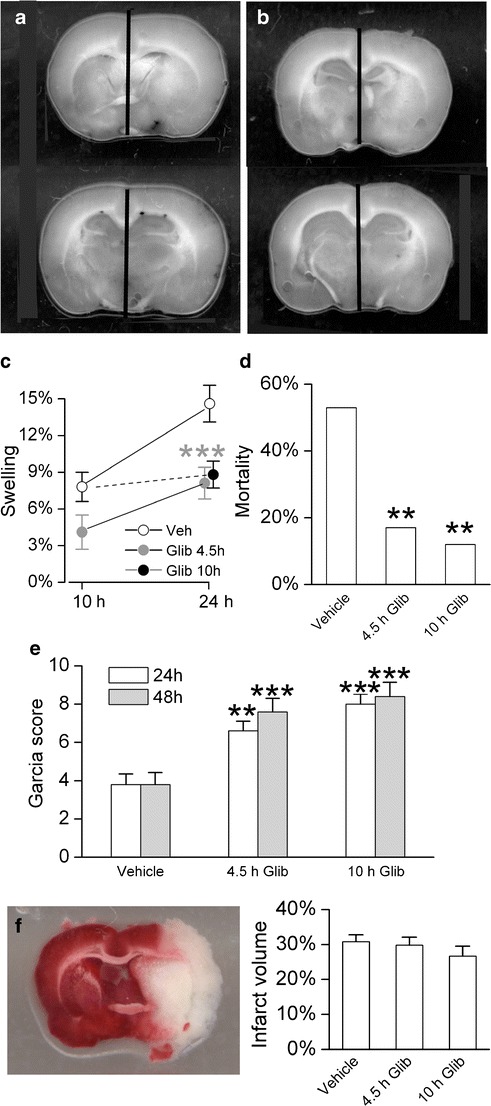
Glibenclamide improves outcomes in a clinically relevant model of stroke. Hemispheric swelling imaged at 24 h in rats administered vehicle (**a**) or glibenclamide (**b**) 4.5 h after onset of ischemia; *vertical bars* denote the location of midline structures; the *scatter plot* (**c**) shows the percent hemispheric swelling at 10 and 24 h in rats administered vehicle (*empty circles*) or glibenclamide at 4.5 h (Glib 4.5 h; *filled gray circles*) or 10 h (Glib 10 h; *filled black circles*) after onset of ischemia; 8–10 rats per group; ***P < 0.001, pertains to analysis of the four groups treated at 4.5 h. **d** Mortality, assessed at 48 h, in rats administered vehicle, glibenclamide at 4.5 h after onset of ischemia (4.5 h Glib) or glibenclamide at 10 h after onset of ischemia (10 h Glib); 51, 41, and 25 rats in the three groups, respectively; **P < 0.01; data at 4.5 and 10 h are not statistically different from each other (*P* = 0.7). **e** Garcia scores, assessed at 24 h (*empty bars*) and 48 h (*gray bars*), in rats administered vehicle, glibenclamide at 4.5 h after onset of ischemia (4.5 h Glib), or glibenclamide at 10 h after onset of ischemia (10 h Glib); same rats as in **d**; **P < 0.01; ***P < 0.001; data at 4.5 and 10 h are not statistically different from each other (*P* > 0.05). **f** Representative infarct visualized using TTC (*left*), and comparison of infarct volumes measured at 48 h in rats administered vehicle or glibenclamide at 4.5 or 10 h after onset of ischemia, as indicated (*P* = 0.5)

Edema and hemispheric swelling were previously shown to be reduced by glibenclamide [21, 23, 25], but not in the model studied here or when administered as late as 10 h after onset of ischemia. First, we measured hemispheric swelling 10 and 24 h after onset of ischemia in rats administered either vehicle or glibenclamide at 4.5 h (Fig. 3a–c). Glibenclamide given at 4.5 h reduced swelling measured at 10 h from 7.8 ± 1.2% to 4.1 ± 1.4%. Glibenclamide given at 4.5 h reduced swelling measured at 24 h from 14.7 ± 1.5% to 8.1 ± 1.3%. Statistical analysis showed that the effects of both time (progression of swelling between 10 and 24 h) and treatment (vehicle vs. glibenclamide) were significant (both *P* < 0.001, by two-way ANOVA).

We also measured hemispheric swelling at 24 h in rats administered glibenclamide at 10 h (Fig. 3c). We used the previous data—vehicle-treated rats assessed at 10 h that exhibited 7.8 ± 1.2% hemispheric swelling—as controls for this experiment. When glibenclamide was administered at 10 h, swelling at 24 h was only slightly more (8.8 ± 1.1%; *P* = 0.56, by *t*-test) than at 10 h without treatment, suggesting that glibenclamide largely arrested the formation of edema during this period of time. When glibenclamide was administered at 10 h, swelling at 24 h was significantly different compared to vehicle-treated rats (*P* = 0.005, by *t*-test).

### Mortality

Mortality was previously shown to be reduced by glibenclamide [21, 23], but not in the model studied here or with a treatment delay of 10 h. Mortality was assessed 48 h after onset of ischemia. For the three groups (vehicle, 4.5 h, and 10 h glibenclamide), mortality was 53%, 17%, and 12%, respectively (Fig. 3d). Values with glibenclamide administered at both 4.5 and 10 h were significantly different from that in the control group (both *P* < 0.01). Values with glibenclamide at 4.5 and 10 h were not statistically different from each other (*P* = 0.7).

### Garcia Scores

Garcia scores were assessed 24 h after onset of ischemia. For the three groups (vehicle, 4.5 h, and 10 h glibenclamide), scores were (mean ± SEM) 3.8 ± 0.55, 6.6 ± 0.51, and 8.0 ± 0.52, respectively (Fig. 3e, empty bars). Values with glibenclamide administered at 4.5 (*P* < 0.01) and 10 h (*P* < 0.001) were significantly different from that in the control group. Values with glibenclamide at 4.5 and 10 h were not statistically different from each other (*P* > 0.05).

Garcia scores were assessed 48 h after onset of ischemia. For the three groups (vehicle, 4.5 h, and 10 h glibenclamide), scores were (mean ± SEM) 3.8 ± 0.62, 7.6 ± 0.70, and 8.4 ± 0.74, respectively (Fig. 3e, gray bars). Values with glibenclamide administered at 4.5 and 10 h were significantly different from that in the control group (both *P* < 0.001). Values with glibenclamide at 4.5 and 10 h were not statistically different from each other (*P* > 0.05).

In vehicle-treated rats that survived 48 h with a Garcia score >1, the Garcia score at 48 h was negatively correlated with the reduction in LDF signal recorded at the onset of ischemia (Spearman *r* = −0.62; *P* < 0.01). By contrast, in rats treated with glibenclamide at 4.5 h, this correlation was lost (Spearman *r* = 0.28; *P* = 0.1). The significant negative correlation in the vehicle-treated group may have been due to continued brain swelling, which would be expected to be worse with a more severe ischemic insult. The loss of correlation with glibenclamide would be consistent with reduced swelling in this group (Fig. 3a–c) [21, 23].

For rats that survived the first 24 h (Garcia score >0), we also examined the changes in Garcia scores that occurred subsequently. In vehicle-treated rats, 4/28 survivors (14%) died between 24 and 48 h, and overall Garcia scores were unchanged (6.9 ± 0.5 and 6.9 ± 0.7 at 24 and 48 h, respectively; *P* = 0.61). In rats treated with glibenclamide at 4.5 or 10 h, 3/59 survivors (5%) died between 24 and 48 h, and overall scores improved between 24 and 48 h, from 8.0 ± 0.2 to 8.9 ± 0.4 (*P* < 0.01).

### Infarct Size

TTC-infarct volumes were assessed 48 h after onset of ischemia (Fig. 3f). For the three groups (vehicle, 4.5 h, and 10 h glibenclamide), corrected infarct volumes were (mean ± SEM): 30.8 ± 2.0%, 29.8 ± 2.3%, and 26.7 ± 2.8%, respectively (*P* = 0.5).

In vehicle-treated rats that survived 48 h with a Garcia score >1, the Garcia score at 48 h was negatively correlated with infarct volume (Spearman *r* = −0.81; *P* < 0.0001). By contrast, in rats treated with glibenclamide at 4.5 h, this correlation was lost (Spearman *r* = −0.34; *P* = 0.06).

### Hemorrhagic Transformation

Confluent areas of hemorrhagic transformation of an ischemic region, as often reported with 10 mg/kg rtPA in rats [14], were not seen with the dose of 0.9 mg/kg used here, but in some rats, discrete petechial hemorrhages, 0.4–1 mm, were observed. For the three groups (vehicle, 4.5 h, and 10 h glibenclamide), petechial hemorrhages were identified in 6/51, 6/41, and 3/25 rats, respectively (*P* = 0.76, 0.99, respectively, compared to control). In all cases, a single petechial hemorrhage was located in the caudate; in two of the rats, a second petechial hemorrhage also was located in the cortex.

### Validation of Drug Delivery System

To confirm proper functioning of the pumps in the preclinical studies reported above, frequent periodic checks were made of the volume of solution remaining in the pump at the time of either stroke-related death or planned euthanasia (48 h). In 50 cases tested, the residual volume was appropriate for the time of removal of the pump from the animal, given the measured volume that was loaded (222–238 μl), the time when priming was started, and the nominal rate of delivery of 1 μl/h. The same samples also were tested to confirm the presence of vehicle (20 pumps) vs. drug (30 pumps), as well as the concentration of glibenclamide in solution. Measurements of glibenclamide solutions removed at ~24 and 48 h showed values of 94 ± 1.3% and 86 ± 1.8% of the expected concentration, respectively (16 and 14 pumps each), which accorded well with the previous measurements in Fig. 1c.

## Discussion

There are two important new findings in the present study. First, our data indicate that glibenclamide is highly effective when administered as late as 10 h after onset of ischemia. Previous work showed efficacy of glibenclamide out to 6 h [23]. However, recent elucidation of the molecular mechanism responsible for transcriptional upregulation of the Sur1-channel [30], combined with the fact that brain swelling after ischemia takes hours to develop [13, 20], suggested that the treatment window for glibenclamide might exceed 6 h. This was confirmed here, with data indicating undiminished efficacy out to 10 h. Previous work with rat models of MCAo showed treatment efficacy (infarct volume or neurological function) for a variety of agents when treatment was initiated at 10 h after onset of ischemia (ischemic insult: 2-h MCAo [28, 29]), at 12 h (ischemic insult: 1-h [32], 1.5-h [10], and 2-h [27] MCAo), and at 18 h (ischemic insult: 20-min MCAo [10]). To our knowledge, no agent other than glibenclamide has shown treatment efficacy on mortality from malignant edema and on neurological function when administered at 10 h in a model with 4.5-h MCAo.

Secondly, in the model used here, glibenclamide appeared to dissociate functional outcomes (mortality and Garcia scores) from infarct volumes. Although previous reports that examined either permanent occlusion [18, 25] or a shorter ischemic time (105 min) [25] indicated that glibenclamide reduces lesion volumes, here lesion volumes were not changed by treatment. The 4.5-h duration of ischemia used here apparently was associated with a maximum ischemic insult when measured at 48 h, leaving little opportunity for a reduction in infarct volume by drug. Many reports indicate that 3 h of mechanical occlusion produce maximum infarct volumes when measured 48 h later, and that longer occlusion times do not increase infarct size further [4, 31]. Similarly, in rat models, administration of rtPA (10 mg/kg) is ineffective in reducing infarct volume when given more than 2–4 h after onset of ischemia [4, 16, 33]. Together, these studies indicate that it should not be surprising that infarct volumes would have been similar in vehicle vs. glibenclamide groups. However, despite the same infarct volumes, functional outcomes were significantly better with glibenclamide, indicating that some factor other than infarct volume determined functional outcome. The other factor likely was edema or brain swelling. Prior studies showed that edema and brain swelling are significantly ameliorated by glibenclamide [18, 21, 23, 25], as was reaffirmed in the present study in a different model of stroke.

An important goal of preclinical stroke studies is the development and use of clinically relevant models that may be helpful in predicting the outcome of clinical trials. Numerous models of ischemic stroke in rats have been described, but few precisely mimic clinical stroke [5]. Truly permanent occlusion, as studied in rats with occluders permanently positioned at the ICA bifurcation, rarely occurs in humans because of eventual spontaneous recanalization. Also, nearly all transient ischemia models studied in rats utilize relatively short ischemia times in order to preclude fatal swelling. In the model we studied, recanalization was obtained by removing the occluder at 4.5 h, simulating intra-arterial mechanical clot retrieval [6]. At the same time, we administered rtPA at the latest time and at the dose used in humans (0.9 mg/kg IV) [2, 9]. Although this dose is expected to be minimally thrombolytic in rats [11], it was important to avoid the excess toxicity associated with 10 mg/kg rtPA [9] in order to maintain relevance to humans. In addition, use of a sub-thrombolytic dose served to mimic the situation frequently encountered in humans wherein administration of rtPA does not lead to timely recanalization or reperfusion [1, 26]. Finally, our outcomes were focused on improvements in mortality, brain swelling and neurological function, rather than a reduction in lesion volume, because of the indisputable clinical impact of the first three—rtPA, currently the only drug approved for use in stroke, was approved based on its demonstrated ability to improve functional outcomes, not because it reduces lesion volumes. These considerations led to the model described here, which we believe to be highly relevant clinically.

An important limitation of the present study is that we did not examine the effect of occluder removal plus rtPA administration at 4.5 h on LDF signals or other measures of cerebral blood flow. As a result, we do not know the status or timing of cerebral reperfusion in this model. In our previous study [23], removing the occluder at 6 h (no rtPA administered) often resulted in delay of reperfusion (judged by LDF signals) by up to 2 h, consistent with development of thrombosis during occlusion, and delayed spontaneous thrombolysis after occluder removal. It is likely that in the model studied here, thrombolysis was eventually achieved, but when this occurred, in which vessels it occurred, and whether it occurred too late to salvage tissues, is not known. Another limitation of the present study is that we did not study survivors beyond 48 h. However, our data indicate that rats administered glibenclamide, but not vehicle, improved significantly between 24 and 48 h. Also, our previous experience with the Garcia scoring system showed that rats exhibiting scores of 6 or better at 48 h rarely die and almost always improve functionally [23]. Thus, it seems unlikely that the beneficial effects of glibenclamide observed here at 48 h would have been negated by longer observation.

In summary, we describe a rat model of stroke that was designed to mimic several aspects of the human condition, including severe ischemia (75% < reduction in LDF signals ≤90%), abrupt recanalization at 4.5 h, administration at 4.5 h of the dose of rtPA used in humans, and evaluation of clinically relevant endpoints. The insult produced, combined with other features of the model, yielded a severe injury with high mortality and poor functional outcome. Using this model, we demonstrated that outcomes (swelling, mortality, and neurological function) were significantly improved when low-dose glibenclamide was administered at either 4.5 or 10 h after onset of ischemia.
